# Wnt Signaling: Pathogen Incursion and Immune Defense

**DOI:** 10.3389/fimmu.2019.02551

**Published:** 2019-10-29

**Authors:** Suborno Jati, Tresa Rani Sarraf, Debdut Naskar, Malini Sen

**Affiliations:** ^1^Division of Cancer Biology and Inflammatory Disorder, Indian Institute of Chemical Biology, Kolkata, India; ^2^Department of Biotechnology, Maulana Abul Kalam Azad University of Technology, Kolkata, India

**Keywords:** Wnt, frizzled, pathogen, microbiota, actin, cytoskeleton, macrophage, immunity

## Abstract

Wnt ligands interact with the transmembrane cell surface receptors Frizzled and ROR/RYK to initiate complex signaling cascades that are crucial for cell physiology and the proper functioning of the immune system. Wnt signaling is instrumental in maintaining immune surveillance and during infections by pathogenic microbes helps mount host resistance to infection. Some pathogens, however, utilize Wnt signaling to build a niche for their survival. The goal of this review is to summarize current and developing concepts about the tug of war between Wnt signaling and pathogens for deployment of host resources, focusing mostly on macrophages and cytoskeletal actin dynamics. An additional objective is to outline the interrelation between Wnt signaling and the host microbiota, which is vital for immune defense, discussing in the same perspective, how Wnt signaling could be differentiating pathogen from non-pathogen.

## Introduction

Macrophages, major sentinels of immune defense utilize the Wnt signaling scheme to sustain immune homeostasis, maintain immune surveillance, and combat infections with several pathogens. Some microorganisms, however, outmaneuver Wnt signaling and exploit it to create a niche for their survival. Wnt associated cytoskeletal modulations and transcriptional programs partake of such host pathogen interactions (1–8). Wnt signaling is also linked with the colonization of distinct groups of microbiota, which coexist with the host and bolster immune defense by inhibiting the growth of pathogenic microbes (9, 10).

Wnt proteins constitute a family of about 19 different secreted cysteine-rich glycoproteins in mammals, which are highly conserved among different species. Nusse et al. first identified the mammalian counterpart of Drosophila Wingless and termed it Wnt1, which nomenclature wise is a combination of Drosophila Wingless and the mouse proto-oncogene Int1 (11–13). The role of Wnt (Wingless) signaling was first documented during Drosophila development (11, 13–15). Subsequently, with the identification of Wnt homologs in mammals the importance of Wnt signaling was recognized in the context of different cellular functions ranging from cell proliferation and migration to cell polarity and tissue homeostasis (16). Wnt signaling initiates when Wnt ligands interact with the Frizzled and ROR or RYK family cell surface receptors. While the Frizzleds (about 12 in number) are transmembrane proteins resembling heterotrimeric G protein coupled receptors, ROR1, ROR2, and RYK resemble tyrosine kinases (12, 17–22). Due to considerable homology among different members of the Wnt and Frizzled families, it is possible for a particular Wnt ligand to interact with multiple Frizzled receptors (23). Thus, the outcome of Wnt-Frizzled signaling depends on the prevailing stoichiometry of the Wnt and Frizzled proteins and their mutual accessibility (24). Wnt-ROR/RYK signaling, mostly described as independent of Wnt-Frizzled signaling has also been shown to support Wnt-Frizzled signaling in some cases (21). A general scheme for Wnt signaling has been described in several review articles (25, 26).

Wnt signaling can be categorized into two main classes—canonical (β-catenin dependent) and non-canonical (β-catenin independent). In canonical Wnt signaling, β-catenin, which accumulates in the cytoplasm, enters into the nucleus acting as a transactivator for LEF/TCF transcription factors to initiate expression of β-catenin responsive genes (27). In non-canonical Wnt signaling, transcriptional activation is mostly associated with transcription factors such as NFκB, NFAT, and AP1 (6, 28, 29), and cell polarity is linked with modulations of the cytoskeleton that involve actin and actin associated proteins (30–32). The ligands Wnt3A and Wnt5A are usually considered as representatives of the canonical and non-canonical modes of Wnt signaling, respectively (33). Since Wnt signaling is guided and controlled by fairly homologous receptors and intracellular signaling intermediates that may be shared by both canonical and non-canonical modes of Wnt signaling, overlap between these two signaling pathways is not uncommon. Quite interestingly, the intracellular adaptor molecule Disheveled and Daple are required for both modes of Wnt signaling (34–36). Disheveled utilizes cholesterol, and the heterotrimeric G proteins coupled to Frizzled receptors interact with Daple for switching between the canonical and non-canonical modes (37). It is important to know if cooperation of the heterotrimeric G proteins is required for Disheveled function during signaling (36, 38). Cell surface co-activator receptors such as Lipoprotein Receptor-like Protein (LRP) 5/6 are usually associated with only the canonical mode of Wnt signaling. Although LRP5/6 has been shown to interact with the non-canonical Wnt signaling ligand Wnt5A (24, 39), whether it is actually required for non-canonical Wnt signaling or acts to strike a balance between the canonical and non-canonical modes remains unsettled. A recent report suggests that Wnt can also signal by binding to TLR4/2 receptors (40). While Wnt-TLR signaling is important in view of the evolving role of Wnts in immunity to infections, the mode of this signaling pathway in relation to the already established intracellular signaling adaptors and intermediates remains to be documented.

Although it is now clear that Wnt signaling is closely associated with infection, sustenance of host microbiota and immune regulation (3–6, 9), interrelations between Wnt signaling and host immunity to different types of infection are complex and the molecular details therein are not yet settled. The goal of this review is to summarize current and developing concepts relating to the role of Wnt signaling in microbial infections with special emphasis on macrophages and cytoskeletal actin. The idea is to address unanswered yet important questions based on existing knowledge and paradigms to help bridge gaps in our understanding of the Wnt signaling scheme in the context of host immune defense programs.

## Interrelation Between Wnt Signaling and Infection With Pathogenic Microbes

Wnt signaling in macrophages plays a crucial role in shaping the outcome of infections by different pathogens (3, 4, 41–43). While Wnt signaling facilitates elimination of certain infections by disabling the causative pathogens, it also favors the progression of other infections by allowing the causative pathogen to build its niche within macrophages. It is expected that such interactions of host Wnt signaling with the encountered pathogens would involve actin cytoskeletal dynamics, an integral component of host pathogen interactions (32, 44, 45). We will focus on some representative microbial infections in this regard.

### Bacterial Infection

Infection by bacterial pathogens such as *Pseudomonas* sp. and *Streptococcus* sp. is inhibited by Wnt signaling. Wnt induced bacterial clearance in macrophages occurs by phagocytosis and subsequent xenophagy through utilization of the host actin associated autophagy circuit. In this context, the function of Wnt5A, which mediates cytoskeletal actin modulations through activation of the actin associated proteins Rac1 and Disheveled holds considerable significance (3, 4, 36). It has been demonstrated that Wnt5A signaling in macrophages not only facilitates internalization of pathogenic *Pseudomonas* sp. and *Streptococcus* sp., but also enhances their killing. Wnt5A-Rac1/Disheveled dependent cytoskeletal actin rearrangements facilitate the formation of bacteria containing autophagosomes that are destined for lysosomal fusion, thus enabling bacterial clearance (3). Rac1 and Disheveled inhibitors, as well as Cytochalasin D which inhibits actin assembly, suppress Wnt5A mediated killing of *Pseudomonas* and *Streptococcus*. Signaling by Wnt3A and Wnt11 in macrophages and macrophage associated cells of the gut has also been linked with inhibition of infections by pathogenic *Pseudomonas* and *Salmonella* species (43, 46). It is important to know if cytoskeletal modulations as observed in the case of Wnt5A signaling are also associated with reduction of bacterial pathogen load by Wnt3A and Wnt11.

The Human Monocytotropic Ehrlichiosis (HME) causing bacterium *Ehrlichia chaffeensis* is yet another bacterial pathogen that interacts with Wnt signaling in macrophages. Unlike some of the other bacteria, *E. chaffeensis* utilizes both so called canonical and non-canonical Wnt signaling intermediates for niche building and multiplication within macrophages. Infection by *E. chaffeensis* is accompanied by increase in activation of the Wnt signaling intermediates Rac1 and Disheveled (8). Blockade in expression of Wnt5A, its putative receptor Frizzled5 and Rac1 furthermore, leads to decrease in *E. chaffeensis* infection (8), suggesting that Wnt5A signaling is needed for promoting *E. chaffeensis* intracellular niche formation. Interestingly, blockade in Wnt5A signaling also inhibits infection by the non-pathogenic lab strain of *E. coli*, DH5-α, which gets internalized but not killed by activation of Wnt5A signaling (5). Wnt5A induced internalization of *E. coli* DH5-α is dependent upon alterations in actin assembly, which are not conducive to bacterial killing (5). Whether similar alterations in actin assembly are also associated with *E. chaffeensis* infection and niche formation will be important to decipher.

Wnt signaling in macrophages also regulates infection by different *Mycobacterium* species. Mycobacterial infections are associated with altered expression of several Wnts (47–51). While Wnt5A has been reported to promote both pro inflammatory and anti-inflammatory cytokine signatures in mycobacterium infected macrophages, both Wnt3A and Wnt6 have been reported to skew the intracellular milieu of infected macrophages toward an anti-inflammatory cytokine signature (47, 49, 52, 53). Recently, Gao et al. has demonstrated that Wnt5A restricts niche formation in macrophages by both *Mycobacterium tuberculosis* and *Mycobacterium bovis* through activation of autophagy (54). In light of the documented role of Wnt5A signaling in cytoskeletal modulations and autophagy / xenophagy (3, 4, 30, 31), and the crucial involvement of actin and actin binding proteins during mycobacterial infections (55–57), it is important to examine how Wnt mediated cytoskeletal modulations regulate the sustenance vs. inhibition of mycobacterial infections.

### Parasitic and Fungal Infection

Several lines of evidence indicate that Wnt signaling in macrophages regulates parasitic and fungal infections. Wnt5A signaling suppresses infection by the parasite *Leishmania donovani*, the causative agent of visceral leishmaniasis by blocking the sustenance of *L. donovani* containing parasitophorous vacuoles within macrophages. Transmission electron microscopy of *L. donovani* infected macrophages has revealed the predominance of degraded parasitophorous vacuoles in autophagosome like intracellular vesicles upon activation of Wnt5A signaling (4). Wnt5A mediated cytoskeletal alterations correlate with parasitophorous vacuole degradation and inhibition of infection. As in the case of bacterial infection, Wnt5A mediated inhibition of *L. donovani* infection is blocked by inhibitor of Rac1 activation, which is linked with Wnt5A mediated cytoskeletal alterations (4). Inhibition of Wnt5A production *in vivo* by intravenous administration of the inhibitor IWP2 into mice accordingly results in increased susceptibility to *L. donovani* infection (4). *L. donovani* infection in mice reportedly is sustained through a skewed hematopoiesis/myelopoiesis program marked by the predominant prevalence of inflammatory monocytes (58–60). Myelopoiesis and macrophage differentiation under normal conditions are supported by a complex Wnt signaling network that includes Wnt5A (6, 61–64). Thus, sustenance vs. inhibition of *L. donovani* infection is perhaps determined by the diverse traits of Wnt signaling in the context of the infection load. It is important to carry out a detailed evaluation of the role of Wnt5A in the context of other Wnts in this respect, especially in light of the documented antagonism of Wnt5A—actin network toward *L. donovani* infection (4).

The parasite *Trypanosoma cruzi*, which causes Chagas disease, unlike *L. donovani* exploits Wnt signaling for survival as is evident from the diminution of the intensity of *T. cruzi* infection through inhibition of transcriptional activation by β-catenin. *T. cruzi* infection increases expression of several Wnt ligands and Frizzled receptors, including Wnt5A, Wnt3A, Frizzled 4, Frizzled 8, Frizzled 9, and Frizzled 6 (7). Detailed analysis of Wnt-Frizzed signaling pathways in relation to *T. cruzi* infection should unveil the molecular mechanism of intracellular niche building by the parasite and the involvement of cytoskeletal actin therein.

Similar to *L. donovani* infection, and unlike *T. cruzi* infection, fungal infection of macrophages by *Aspergillus fumigatus* is inhibited by Wnt5A signaling. Migration and accumulation of neutrophils caused by Erk1/2 and JNK mediated increase in production of Wnt5A after Dectin1/Lox1 assisted intracellular entry of the fungus have been identified as a cause for the fungal clearance (65). It is important to examine if secreted Wnt5A and actin alterations are directly involved in recruiting neutrophils and activating macrophages for phagocytic clearance of *A. fumigatus*.

### Overview of Wnt—Pathogen Interactions

In summary, it appears from the cited examples of different host pathogen interactions that the effect of Wnt signaling on pathogen infection is dependent on the type of the pathogen. Table 1 summarizes the different interrelations between Wnt signaling and pathogen infections. Although *Pseudomonas* sp. */Streptococcus* sp., *L. donovani* and *A. fumigatus* are genotypically dissimilar and exercise different modes of action to infect the host (66–68), activation of Wnt5A signaling inhibits initiation of infection by all of these pathogens independent of their genotypic differences perhaps by inducing alterations in actin assembly which are incompatible with pathogen survival. Wnt mediated cytoskeletal alterations may also be crucial for the regulation of infection by *T. cruzi* and *Mycobacterium*.

**Table 1:** Wnt signaling and host-pathogen interaction.

**S.no**.	**Pathogen**	**Disease**	**Associated Wnt homolog**	**Host-pathogen interaction**	**References**
1.	*Pseudomonas* sp., *Streptococcus* sp.	Respiratory diseases (e.g., COPD, sepsis etc.)	Wnt5a, Wnt3A	Wnt5a-RAC1-Disheveled mediated cytoskeletal actin rearrangement facilitates autophagy and containment of infection.	(3)
				Wnt3A mediated increase in antimicrobial peptides causes killing of *Pseudomonas*.	(46)
2.	*Salmonella* sp.	Inflammatory bowel disease (IBD), Typhoid	Wnt11	Wnt11 signaling protects the host from bacterial infection and inhibits apoptosis in intestinal cells.	(43)
3.	*Ehrlichia chaffeensis*	Human monocytotropic ehrlichiosis (HME)	Wnt5a, Wnt10, Wnt6	Wnt ligands and associated signaling pathway (β-catenin mediated, NFAT-C1 mediated and others) promotes survival of the pathogen inside the host.	(8)
4.	*Mycobacterium tuberculosis*	Tuberculosis	Wnt5a, Wnt3A, Wnt6	Mycobacterium infection promotes Wnt5A expression in human PBMC and blockade of Wnt5A signaling results in inhibition of IL-12p40 and IFNγ secretion. Mycobacterium infection downregulates Wnt5A expression in mouse lungs.	(47, 50)
				Enhanced IL36γ secretion during infection induces Wnt5A expression which aids in controlling infection through COX-2 mediated autophagy.	(54)
				Wnt3A promotes an anti-inflammatory effect in murine macrophages during infection in lungs.	(49, 52, 53)
				Mycobacterium infection induces Wnt6 expression and promotes anti-inflammatory phenotype of macrophages through Arginase-1 expression.	(47)
5.	*Leishmania donovani*	Visceral leishmaniasis (Kala azar)	Wnt5a	Wnt5A-Rac1-Rho mediated cytoskeletal alteration promotes enhanced fusion of parasitophorous vacuole with lysosome which helps in restraining infection.	(4)
6.	*Trypanosoma cruzi*	Chagas disease	Wnt3a, Wnt5a	*T. cruzi* early infection increases expression of Wnt5A, 3A, several Frizzled receptors, and Wnt signaling intermediates.	(7)
				Activation of β-catenin promotes inhibition of inflammatory cytokine secretion and replication of parasite.	
7.	*Aspergillus fumigatus*	Fungal keratitis	Wnt5a	Host PRR activates Wnt5A expression through ERK and JNK pathway. Wnt5A attracts neutrophils for clearance of *Aspergillus fumigatus*.	(65)

In light of reports of existing links between cytoskeletal modulation and transcription factor translocation in immune cells (69, 70), it remains to be seen if activation of immune response associated transcription factors such as NFκB, NFAT, or AP1 correlates with cytoskeletal actin alterations during pathogen clearance facilitated by Wnt signaling. Wnt5A signaling, nevertheless, has been described in separate studies to sustain nuclear translocation of NFκB in macrophages both in the steady state and in response to mycobacterial infection, thereby maintaining expression of NFκB responsive immune response genes as a potential means of host resistance (6, 50). The requirement of NFκB mediated gene transcription for immunity to bacterial infections has, furthermore, been independently documented (71–73). Wnt5A signaling can also influence transcriptional regulation of immune response genes such as TNFα, IFNγ, and IL6, which have interrelation with actin dynamics and are known to work toward inhibition of pathogen infection (2, 5, 6, 74–77). It will be interesting to find out how cytokine associated actin dynamics directly links with inhibition of pathogen infection.

Cytoskeletal alterations and transcriptional networks associated with the interaction of pathogens with different degrees and types of Wnt signaling are clearly complex. Careful analyses of further in depth studies with special attention to the different study approaches used by different laboratories are needed in order to establish how the different Wnt molecules work to accommodate or annihilate different pathogens through diverse signaling pathways.

## Interrelation Between Wnt Signaling and the Host Microbiota

Several anatomical locations in the human host serve as home to distinct congregations of bacteria (microbiota) that synchronize with host immune programs against infections (78–81). In this context, the microbiota of the gut deserve special mention on account of their prominent prevalence in several niches of the gut especially the Peyer's patches and the *lamina propria* (82). These microbiota exist both in the gut lumen as well as in close proximity with different kinds of macrophages and contribute to immune regulation in the host. For instance, although not clearly understood how, gut microbiota are required for the maintenance of sIgA and a steady state cytokine milieu in the gut lumen, which can potentially serve to fight off pathogens (78, 83, 84). Gut microbiota are also known to secrete antibacterial peptides, which are required for immune defense (10, 85).

Wnt signaling is important for gut organogenesis (86, 87) and sustains intestinal homeostasis through maintenance of specific microflora. Several lines of evidence suggest that colonization of microbiota in specific gut regions correlates with differential expression of Wnts such as Wnt5A and Wnt3A, and Wnt signaling intermediates for example β-catenin (9). Wnt signaling components such as Axin and Disheveled on the other hand, have been shown to act synergistically with Synbindin, a syndecan-2 binding protein, to influence gut microbiota composition (88). How the apparent symbiosis between Wnt signaling and gut microbiota composition relates to immune defense, however remains undocumented. In view of the fact that Wnt signaling, in particular Wnt5A signaling facilitates internalization and destruction of several pathogens through its influence on the cytoskeletal dynamics and autophagy machinery of the host macrophage (3, 4), but coordinates with the resident host microbiota (9), it is quite evident that Wnt signaling is able to differentiate pathogen from non-pathogen. But, how does this happen? Although there is evidence that some microbiota are present within the resident phagocytes (89, 90), it is unclear if the microbial niche is created or promoted by Wnt signaling and how it relates to the cytoskeletal actin network. It also remains to be seen if host resident microbiota influences the phagocytosis and clearance of pathogenic microbes and whether such interrelation involves Wnt signaling.

## Conclusion and Future Directions

Complex Wnt signaling schemes that are intrinsically associated with macrophage mediated immune functions (e.g., phagocytosis, autophagy/xenophagy) conform to the in-built maneuvering of macrophages as they confront with different kinds of pathogens (1, 91). Several lines of evidence substantiate that Wnt signaling, in particular Wnt5A signaling, is important for the cytoskeletal modulations and transcriptional programs inherent to macrophages during immune surveillance (3, 4, 6). While many pathogens are disabled through activation of Wnt5A signaling, some pathogens utilize it for their own survival within the host macrophage (3, 4, 7, 8, 65). Moreover, although not known exactly how, colonization of distinct microbiota within the lumen and macrophage associated niches within the gut and other anatomical locations correlate with differential expression of different Wnts and their signaling intermediates (9). The different nuances of Wnt signaling that annihilate some microbes, yet allow the growth and proliferation of others, at the same time sustaining the colonization of diverse microbiota, remain largely uncharacterized. In this regard one may come up with several explanations. In light of the fact that Wnt5A signaling alters actin assembly (3, 4, 30), a vital component of host pathogen interaction, it is possible that Wnt5A induced alterations in actin assembly influence different pathogens differently, depending on the nature of virulence factors, thereby either inhibiting or facilitating their survival. The situation perhaps is guided by the extent of Wnt5A signaling in macrophages, as we observed inhibition of infection by pathogenic microbes through activation of Wnt5A signaling and increased infection through its blockade (3, 4). The decision for pathogen destruction vis. a vis. survival is possibly based on the nature of the tussle between host induced and pathogen induced modulations (conformations) of actin and actin binding proteins in the very early stages of infection wherein host Wnt5A signaling plays a fundamental role. In this connection, knowledge of the status of the macrophage associated resident microbiota, in relation to Wnt signaling and cytoskeletal dynamics is vital.

In future, the influence of Wnt signaling on actin modulations at the initial stages of infection needs to be deciphered at the molecular level in the context of different microbes, ranging from virulent pathogens to the resident microbiota, which may be deemed as non-pathogens. Additionally, the potential links between the cytoskeletal actin dynamics and transcriptional programs need to be carefully assessed. In this connection, a good understanding of the cytokine milieu that correlates with the interaction of Wnt signaling with different microbial infections will be important, especially in view of its connection with the different stages of sepsis associated with pathogenic infections (92). A thorough evaluation of Wnt signaling in the context of microbial pathogenesis and colonization of resident microbiota may lead to the development of new modes of therapeutic interventions for the drug resistant refractory microbial infections.

## Author Contributions

MS organized the layout of the article and wrote the article. DN contributed to the layout organization and writing. SJ and TS worked on the table and references and assisted in writing.

### Conflict of Interest

The authors declare that the research was conducted in the absence of any commercial or financial relationships that could be construed as a potential conflict of interest. The reviewer JM declared their collaboration with one of the authors MS as co-editors in the Research Topic and confirmed the absence of any other collaboration to the handling editor.
